# Nutrition Effects of a Family-Centered Health Promotion Program for Mexican-Heritage Children in the Lower Rio Grande Valley of Texas

**DOI:** 10.3390/nu15071600

**Published:** 2023-03-25

**Authors:** Chelsey Laviolette, Cassandra M. Johnson, J. Lauren Butler, Lesli Biediger-Friedman, Joseph R. Sharkey

**Affiliations:** 1 Nutrition and Foods Program, School of Family and Consumer Sciences, Texas State University, 601 University Drive, San Marcos, TX 78666, USA; 2 School of Public Health, Texas A&M University, 212 Adriance Lab Rd., College Station, TX 77843, USA

**Keywords:** border community, Latino/a children, promotora model, nutrition intervention, family-centered approach, reflection spectroscopy

## Abstract

Systemic and social factors, like poverty and food insecurity, negatively influence fruit and vegetable (FV) intake and body mass index (BMI) among Latino/a children. Behavioral programs are needed to support children’s nutrition. This study examined program effects on FV intake and BMI outcomes for Mexican-heritage children (9–11 years). The program used a modified stepped-wedge design in the Lower Rio Grande Valley of Texas (2019 and 2020). *Promotoras* led experiential nutrition education sessions and collected height, weight, and instant skin carotenoid scores (biomarker for FV intake) at pre-test (baseline), post-test (6 weeks), and maintenance (3–4 months after post-test). Mean changes and group differences in skin carotenoid scores, BMI z-scores and percentiles were obtained from analyses of variance. Linear mixed-effects models were used to determine overall program effects. Mexican-heritage children were enrolled (n = 57 and 52.6% female). An overall decrease in skin carotenoid scores was observed at post-test (−15.1; 95% CI: −24.95, −5.33). While scores varied widely (range: 17–498), an increase of 14.8 ± 23.8 points occurred in one intervention group. Compared to the control period, greater reductions in BMI outcomes occurred during the program. These findings provide evidence for the use of strengths-based approaches in behavioral nutrition programs.

## 1. Introduction

Systemic, structural, and social factors, like poverty and food insecurity, negatively influence nutrition for Latino/a children living in border communities [1,2,3,4]. Sharkey et al. determined that only 28% of the children residing in colonias in the Lower Rio Grande Valley of Texas along the Mexico border consumed the recommended amount of calcium, 10% for dietary fiber, 6% for sodium, while none of the children met the recommendation for vitamin D and potassium [1]. Additionally, children with very low food-security status consumed higher total energy, calcium, and percentage of calories from added sugar.

There is an opportunity for health promotion to support nutrition and physical activity for Latino/a children living in limited-resource households and rural communities, including Texas–Mexico border communities, but few nutrition interventions or programs have been designed, implemented, or evaluated for this population. Exemplar interventions for Latino/a children include Crespo et al., Davis et al., and Barragan et al. [5,6,7]. Prior research has documented the importance of strengths-based and culturally relevant approaches for experiential nutrition education interventions or programs [8]. Diaz et al. stated that culturally appropriate information, along with consideration of healthy traditional behaviors and the importance of peers’ opinions, must be considered when developing an intervention to improve health promotion [9]. Lukas et al. stated that, when done through a culturally meaningful way, hands-on cooking classes that highlight learning among peers, could be effective [10]. Additionally, there is growing literature emphasizing the potential of family-centered approaches, within child obesity research, targeting the family as a system [11], the home environment or family dynamics between family members.

To date, there have been no family-centered behavioral nutrition programs for Latino/a children living in border communities. The ¡*Haz Espacio para Papi!* (HEPP, Make Room for Daddy!) program was a novel and theory-based program for Mexican-heritage families living in border communities in the Lower Rio Grande Valley of Texas [12,13,14]. Specifically, HEPP was a father-focused, family-centered program to support nutrition and physical activity [13], and based on the knowledge of the authors, the first program of its kind. The purpose of this manuscript is to examine the effects of the HEPP program for Mexican-heritage children on (1) dietary intake of fruits and vegetables evaluated with the instant skin carotenoid score via the Veggie Meter^®^ and (2) body mass index (BMI) evaluated with age- and sex-adjusted BMI percentiles and BMI z-scores. Findings from this study are an important step in designing culturally relevant and sustainable programs for Mexican-heritage children living in border communities.

## 2. Materials & Methods

### 2.1. Overview, Study Design, and Context

**Figure 1:**
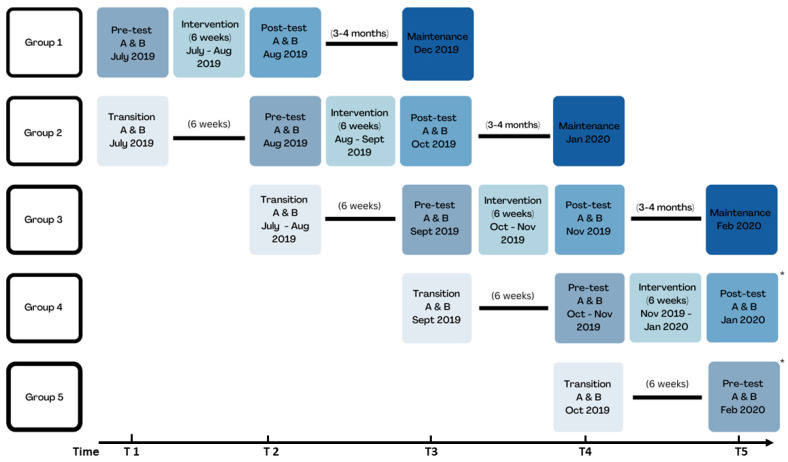
Modified stepped-wedge study design for HEPP program. This figure shows the modified stepped-wedge study design with data collection time points T1 through T5. The transition measure was critical for the delayed intervention (or wait listed control) group. Due to the COVID-19 pandemic in early 2020, group 4 did not complete a maintenance measure (last measure was post-test). Group 5 did not complete the program and had no post-test or maintenance measure (last measure was pre-test).

This manuscript reports on an outcome evaluation of the six-week HEPP (Haz Espacio para Papi | Make Room for Daddy!) program. HEPP utilized a pre-test/post-test (or “pre-post”) design, specifically a modified stepped-wedge design with a comparison wait-listed control or delayed intervention group (Figure 1). The ¡Haz Espacio para Papi! (HEPP, Make Room for Daddy!) nutrition program was conducted between July 2019 and February 2020 in the Lower Rio Grande Valley of Texas [12,13,14]. Participants were Mexican-heritage fathers, mothers, and children. Outcomes were assessed at transition, pre-test (baseline), post-test, and maintenance (short-term follow-up). A transition measure was key for the modified stepped-wedge study design and defined the control/comparison group (see Figure 1 for the modified stepped-wedge study design). *Promotoras* completed the pre-test within two weeks of the program start, completed post-test within two weeks of the program conclusion, and the maintenance within three to four months after post-test. During this time, the COVID-19 pandemic occurred and there were disruptions to program implementation and evaluation. The HEPP program was developed by an interdisciplinary and multi-institutional team. The team consisted of academic-based public health researchers, who had experience and training in nutrition, behavioral and social health, and physical activity, a licensed family psychologist who had expertise in working with Latino family systems, and *promotoras* that received training and had experience in education, research, and social work. *Promotoras* played a critical role in the design, implementation, and evaluation [12,13], as part of community-based participatory research [15,16]. The program was created in both English and Spanish and included an iterative process of development and review. A separate article describes the rationale and design for the HEPP nutrition program in detail [12].

### 2.2. Participants

Prior to recruitment, all materials were approved by the Institutional Review Board (IRB) at Texas A&M. Additionally, collaborating institutions reviewed and approved applications for data analyses. The proposed analyses were submitted and approved by the Texas State University IRB on 27 August 2021 (IRB Protocol #7973). The HEPP program purposefully recruited Latino fathers and mothers. *Promotoras* and community partners helped to recruit families through flyers, word of mouth, and going door to door. As recommended by Panter-Brick et al. and others, the *promotoras* verbally highlighted the uniqueness of the HEPP program and the importance of it to fathers and the whole family [17,18,19,20,21,22].

Eligible parents had to self-identify as Mexican-heritage (self, parent, or grandparent, who was born in Mexico) and be 21 years old or older. Parents also needed to live with their spouse/partner and child (aged 9 to 11 years old), be willing to complete in-person measurement visits at their home for both the pre- and post-test measurements and commit to participate for the full six weeks. Parents and children were excluded from participating if they disclosed having a severe food allergy or physical activity restrictions [3,23,24].

The HEPP program recruited 10 to 12 families from each cluster of colonias (neighborhoods), which were geographically defined within the study area in the Lower Rio Grande Valley of Texas. Program groups were defined based on the neighborhood clusters, and this program recruited from five clusters of colonias (five groups). One group of 10–12 families participated in the program at a time. Each family had one child participating in the program. Random assignment was used to determine which group would start as the intervention (treatment) group and as the wait-listed control.

Development of the informed consent process was done in collaboration with the research team, the *promotoras*, and literature. A Spanish and English informed consent form was developed at or below a fifth-grade reading level with visual aids and graphics to help participants understand the program commitment, research activities, potential risks, and potential benefits. After parental consent, eligible children were asked for their assent to participate in the program. Fathers, mothers, and children received $100 for pre-program visits, $200 for post-program visits, up to $200 for attending all six sessions ($500 per family/household), and a kitchen (food preparation and cooking) kit worth $100.

### 2.3. Program Design

A separate article describes the theoretical foundation and provides a conceptual framework for the HEPP program [12]. Briefly, the Family-centered Action Model of Intervention Layout and Implementation approach (FAMILI) [11], Family Ecological Model (FEM) [25], the Family Systems Theory (FST) [26], and Social Cognitive Theory (SCT) [27] were used to develop a unique theory of action and support multi-level behavior changes at the home or household environment, family system, and individual levels. In addition, formative work informed program development. A field-based research team identified, mapped, and ground-truthed geographic clusters or areas to prepare for the program. Fathers, mothers, and children engaged in independent activities in focus groups, participant-driven photo-elicitation interviews, dyadic interviews, and household elicitation surveys. A separate article describes the formative work in detail [13].

#### Intervention Structure, Components, and Curriculum

HEPP was a six-week program that consisted of three intervention components: weekly in-person group sessions, check-ins (home visits and phone calls), and at-home activities that occurred between the group sessions [12]. The program’s overall theme was embracing existing health-promoting traditions while encouraging new healthy traditions with families. *Promotoras* led in-person group sessions that included a food and beverage tasting for participants with a “mini” nutrition education lesson, an interactive lesson for participants to gain knowledge and skills related to nutrition and family functioning, child-focused cooking lesson, and eating together lesson. *Promotoras* also led weekly check-ins (home visits and phone calls). The nutrition curriculum focused on embracing traditional and cultural foods and food preparations while learning new ways to enjoy beverages, snacks, sides, and main dishes. Every week, a new “spotlight” fruit or vegetable was featured, which included garbanzo bean (chickpea), jicama, cabbage, spinach, sweet potato, and avocado. Most recipes required no animal proteins and were plant-based or vegetarian preparations [12]. A separate article reports on the physical activity curriculum [14].

### 2.4. Data Collection

A team of *promotoras* collected all data simultaneously in person at the homes of participating families [12,13]. Different *promotoras* collected data privately from each family member during the visit. They (*promotoras*) collected data using several techniques: interviewer-administered surveys (sociodemographic data including food security), accelerometry (activity behaviors, including sedentary and physical activity), anthropometry (measured height and weight), and reflection spectroscopy with the Veggie Meter^®^ to obtain instant skin carotenoid score, which is a biomarker for dietary fruit and vegetable intake.

This study was affected by the COVID-19 pandemic. Originally, the study planned assessments at transition (key for modified stepped-wedge study design), baseline or pre-test (within two weeks of program start), post-test (within two weeks after program completion), and maintenance (three to four months after post-test) for all groups (see Figure 1 for the study design). However, the HEPP program stopped in February of 2020 due to the pandemic (and restrictions on in-person contact) and participants in each group did not complete the same set of assessments. This meant that the study canceled group 4 maintenance measures (maintenance measures completed only for groups 1, 2, and 3) and group 5 did not complete the program or any measures after the pre-test. Group 5 completed transition and pre-tests measures only. Program outcomes were assessed at each time point: transition (for groups 2–5), pre-test (all groups, 1–5), post-test (for groups 1–4), and maintenance (for groups 1–3). Table S1 presents data on program completion.

### 2.5. Measures

#### 2.5.1. Food Insecurity

According to the U.S. Department of Agriculture, food insecurity is defined as limited or uncertain availability of nutritionally adequate and safe foods or limited or uncertain ability to acquire acceptable foods in socially acceptable ways [28]. The *promotoras* collected data about food insecurity in an interviewer-administered survey at pre-test and post-test. Food insecurity was assessed based on the validated Hunger Vital Sign two-item food insecurity screener by Hager et al. [29].

#### 2.5.2. Instant Skin Carotenoid Score

The Veggie Meter^®^ is a portable, non-invasive instrument that obtains an objective measure of dietary intake of fruits and vegetables by assessing skin carotenoid levels [30]. The device works through a validated reflection spectroscopy (RS) approach, allowing for carotenoid concentrations in the skin to be objectively measured [30]. The device includes a RS device and a laptop. A Veggie Meter^®^ score has been suggested to reflect fruit and vegetable intake over at least two months (eight weeks) prior [30]. Several validation studies have provided evidence for using the Veggie Meter^®^. Skin carotenoid levels measured by an RS device are an accurate and validated way to measure plasma carotenoid concentrations in children and adults [31,32,33,34]. Jilcott Pitts et al. examined the validity of the RS device between skin and plasma carotenoid concentrations in four different racial and ethnic groups and found a correlation of 0.71 [35]. Furthermore, skin carotenoid concentrations were associated with plasma carotenoid concentrations when adjusted for age, sex, racial/ethnic group, and BMI [35]. Similarly, Jahns et al. found a strong correlation at baseline and moderately strong correlation for skin and plasma carotenoid concentrations across the year [31]. Evidence has shown that the Veggie Meter^®^ is promising for use with individuals across a range of skin tones, and the device automatically adjusts for differences in melanin [30,32].

For this study, the team acquired two Veggie Meter^®^ devices in the summer of 2018 and started training, withs limited information on best practices for Veggie Meter^®^ use. Instructions outlined the importance of calibration with dark and white reference sticks before use, re-calibration at least every two hours during continuous use (not needed for this study, because measures were done with one family per visit), handwashing before assessment, and completing a triple scan on the same finger to obtain an average instant skin carotenoid score. Given the nature of data collection in semi-rural communities like the colonias, the *promotoras* traveled with a portable table for the RS device and laptop, sanitizing wipes, and paper towels to complete the Veggie Meter^®^ assessments. *Promotoras* using the Veggie Meter^®^ followed a study protocol that included traveling with a fully charged Veggie Meter^®^ laptop, calibration before each home visit, setting up the device on a portable table, and ensuring that participants washed hands before assessment. Handwashing helped to remove residual staining from any highly pigmented foods or remnant splotches from colored markers or paint [29]. The *promotoras* used the average setting (versus relying on a single scan), which meant that each child completed three consecutive scans of the same finger to improve reliability [32]. *Promotoras* completed Veggie Meter^®^ scans at each time point (transition, pre-test, post-test, and maintenance) and documented which finger was scanned and any comments regarding protocol deviations. Table S2 reports protocol deviations with the Veggie Meter^®^, out-of-range scores, and the range of scores at each measurement visit.

#### 2.5.3. Children’s Body Mass Index (BMI)

Body Mass Index (BMI) was assessed using age- and sex-adjusted BMI percentile and BMI z-scores based on the 2000 CDC (Centers for Disease Control and Prevention) BMI-for-Age Growth Charts [36]. The CDC recommends using age-adjusted BMI for children, since their height and weight change as they grow, and they also recommend sex-adjustment since body composition varies for boys and girls. The BMI percentile can be interpreted as the relative position of a child’s BMI to children of the same sex and age from the reference population [36]. *Promotoras* obtained measured height (in inches) and weight (in pounds) with a portable stadiometer and digital scale at each time point (transition, pre-test, post-test, and maintenance). They followed a recommended protocol for obtaining height and weight measures and completed three consecutive measurements of height and weight to improve reliability [37]. Average weight was calculated from the multiple weight measurements, and the same procedure was used for height. Children’s BMI was calculated based on the SAS Program macro for the 2000 CDC BMI-for-Age Growth Charts [36]. Data on sex, age (in months), weight (in kg), and height (in cm) were used to calculate children’s age- and sex-adjusted BMI percentiles and z-scores [36].

#### 2.5.4. Data Analysis and Interpretation

Given that the HEPP nutrition program was a pilot study, there was no *a priori* sample size calculation to determine the minimum number of children in the treatment (or intervention) group. This manuscript used all available data from the children who participated in the HEPP program. Of the 59 children enrolled, *promotoras* completed measures at pre-test for 57 children, post-test for 42 children, and maintenance for 24 children. Like prior studies, analyses focused on determining within-person changes in instant skin carotenoid score and BMI outcomes overall, by group and examining group differences [38]. To determine within-person changes in outcomes during the intervention period, changes from pre- to post-test (change_post_ = post–pre) and from post-test to the end of the maintenance period (change_maintenance_ = maintenance–post) were calculated. To determine within-person changes in outcomes during the control period, changes from the transition period to pre-test were calculated (change_control_ = transition–pre). When each group underwent the intervention, the subsequent group served as the preceding group’s control. For example, when group 2 underwent the intervention, group 3 served as the control for group 2. Changes in group 2 skin carotenoid scores between pre- and post-test were compared to changes in group 3 skin carotenoid scores between transition and pre-test. Figure 1 presents the sequence of the measures in the modified stepped-wedge study design.

All data were analyzed using SAS (SAS Institute Inc., SAS Statistical Software: version 9.4. Cary, NC, USA: SAS Institute Inc.) and Stata (StataCorp. Stata Statistical Software: Release 17. College Station, TX, USA: StataCorp LLC). Unadjusted descriptive analyses were used to determine overall and group means for child age in years, instant skin carotenoid score, BMI z-score, BMI percentiles and group distributions by sex, BMI percentile categories and food insecurity status at pre-test measure. Fisher’s exact tests were used to determine differences in the distributions of categorical variables and analysis of variance (ANOVA) was used to test differences in means of continuous variables. To obtain mean pre- to post-test and maintenance to post-test changes for each outcome, six unadjusted ANOVAs were conducted with each within-person change outcome as the dependent variable and group as the independent variable. To determine whether mean within-person changes in each outcome differed by group, three separate ANOVAs were conducted with each within-person change outcome as the dependent variable and an interaction of data collection time point and group as the independent term (time x group). These analyses compared changes in outcomes from pre- to post-test for intervention groups to the changes from transition to pretest for the control groups. Additionally, changes in outcomes from post-test to maintenance for intervention groups were compared to the changes at post-test measures for the control groups. Stata’s margins command was used to obtain unadjusted mean within-person changes in instant skin carotenoid scores, BMI z-scores and BMI percentiles overall and by group from each ANOVA. Linear mixed-effects models were used to determine the overall intervention effects on changes in instant skin carotenoid scores, BMI z-scores and BMI percentiles. Mixed models account for the hierarchical structure of the data, are recommended for use with a stepped-wedge study design [39,40], and allow for the analysis of partial datasets with dropouts or missing study visits. Each model included a fixed effect for child: age in months, sex, month of pre-test data collection, number of intervention sessions attended and baseline instant skin carotenoid score (when change in instant skin carotenoid score was the outcome), baseline BMI z-score (when change in BMI z-score was the outcome), or baseline BMI percentile (when change in BMI percentile was the outcome). Random effects for the assigned study group were included in the model to account for non-independence of members in the same intervention group as either a random intercept or random slope. Results were considered statistically significant at *p* < 0.05. Prior studies were used to interpret results from analyses.

## 3. Results

### 3.1. Sample Characteristics

**Figure 2:**
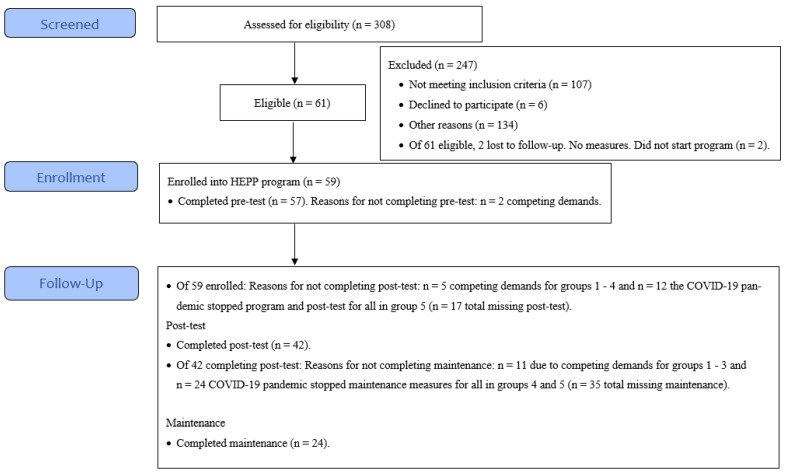
Participant recruitment and enrollment for the HEPP program. Figure shows flow of participants from recruitment through enrollment and follow-up (post-test and maintenance measures).

*Promotoras* screened 308 families for eligibility in the HEPP program, and 61 families met eligibility requirements. Families were recruited from five geographic clusters of neighborhoods (colonias). Two families dropped out before pre-test measures and the program enrolled 59 families or 59 children. Figure 2 shows the flow of children from recruitment and enrollment through program completion and evaluation. This figure was based on the CONSORT (Consolidated Standards of Reporting Trials) flowchart [41].

Table 1 presents characteristics for the analytic sample of children who completed pre-test measures (n = 57). All children were of Mexican heritage (average age 10 years old). Individual and household characteristics were similar across groups. Overall, the sex distribution was 52.6% female and 47.4% male children; however, the distribution varied between some groups. For example, groups 1 and 2 were mostly female children (58.3% and 70.0%, respectively) while group 3 was mostly male children (75.0%). Most children reported household food insecurity at baseline (68.4%).

The program dose delivered was 900 h for the child (six sessions at 2.5 h per session). The mean number of sessions attended was highest for group 1 (5.6 sessions); on average other groups had between 4.7 and 5.4 sessions (Table 2 and Table 3). Unfortunately, the COVID-19 pandemic negatively affected the HEPP program. Program completion is shown in Table S1. Before the pandemic, groups 1–3 completed the program and all measures (pre-test, post-test, and maintenance). However, because of the timing of the pandemic onset in the U.S., group 4 did not complete maintenance measures. Group 5 started the program (first two sessions), but participants did not finish the six-week program (Figure 2). Given the timing of the COVID-19 pandemic, 31 out of 59 children (52.5%) completed all six sessions (Table S1).Retention varied by group. For example, group 1 retained 58.3% of children through session 6 (attended all six sessions). Group 2 had the lowest retention (50%). Group 3 retained proportionally more children than groups 1 or 2 but less than group 3 (69.2% completed all six sessions). Group 4 had the highest retention (83.3%) (Table S1).

### 3.2. Within-Person Change for Instant Skin Carotenoid Scores and BMI for Children at Post-Test and Maintenance

Table 2 presents the unadjusted within-person changes for instant skin carotenoid scores and BMI outcomes for children at post-test overall and by group. Skin carotenoid scores varied widely (range: 17 to 498 across all measurement visits and all children, see Table S2). The only statistically significant difference between groups was for dose, given group 5 stopped the program early due to the COVID-19 pandemic. Overall, the average change for instant skin carotenoid score was −15.3 ± 6.2 (total for all intervention groups), which meant that the instant skin carotenoid score decreased between pre- and post-test for groups 1 through 4. There was no post-test data for group 5 because of the timing of the COVID-19 pandemic. The overall average change for control groups was −5.5 ± 4.2. Notably, group 1 had a 14.8 ± 23.8 score increase between pre- and post-test, while decreases in scores between pre/post-test were observed for groups 2 (−18.2 ± 23.8), 3 (−30.5 ± 21.6), and 4 (−21.7 ± 20.6). Group 3 had the most noticeable change in instant skin carotenoid score (intervention: −30.5 ± 21.6 versus control: −13.4 ± 20.6).

**Table 2:** Descriptive statistics for within-person change in child nutrition outcomes at post-test for Mexican-heritage children.

Outcomes	Total	Group 1	Group 2	Group 3	Group 4	Group 5	*p*-Value
Number of children, *n*	55	9	10	13	12	11	
**Program period**							
Program dose, mean number of sessions (SD)	4.72 (2.15)	5.59 (0.94)	4.69 (2.19)	4.94 (2.12)	5.42 (1.69)	1.27 * (0.79)	<0.001
Within-person change in instant skin carotenoid score	−15.3 (6.2)	14.8 (23.8)	−18.2 (23.8)	−30.5 (21.6)	−21.7 (20.6)	-	
BMI							
Within-person change in BMI percentile	−0.34 (0.4)	1.79 (1.74)	1.43 (1.74)	−3.27 (1.57)	−0.56 (1.51)	-	
Within-person change in BMI z-score	−0.03 (0.02)	0.07 (0.07)	0.04 (0.07)	−0.15 (0.06)	−0.03 (0.06)	-	
**Control period**							
Within-person change in instant skin carotenoid score	−5.5 (4.2)	4.9 (22.6)	−6 (20.6)	−13.4 (20.6)	−6 (21.6)	-	0.695
Within-person change in BMI percentile	0.44 (0.41)	0.66 (1.65)	1.33 (1.51)	−0.58 (1.51)	0.4 (1.57)	-	0.787
Within-person change in BMI z-score	0.03 (0.02)	0.02 (0.07)	0.08 (0.06)	−0.01 (0.06)	0.01 (0.06)	-	0.561

BMI: Body Mass Index; SD Standard Deviation. Data for 55 children excluding four children with missing outcome data at all four data collection time points. This table presents changes in outcomes between the pre-test (baseline) and post-test measures for intervention groups. *Promotoras* completed post-test measures six weeks after baseline. *p*-values are for uncorrected overall *p*-value for analysis of variance (ANOVA) for differences in mean changes between intervention and control groups. * Statistically significant difference between group 5 and all other groups based on *p*-values < 0.05 for group differences generated from Tukey test to control Type 1 error rate.

Regarding BMI outcomes at post-test, the average BMI percentile change was −0.34 ± 0.4 (total for all groups), which meant that BMI percentile decreased between pre- and post-test. The overall average change for control groups was an increase of 0.44 ± 0.41 for BMI percentile. Group 3 had the most noticeable decrease in BMI percentile (intervention: −3.3 ± 1.6 versus control: −0.58 ± 1.5) at post-test. A similar pattern was seen for BMI z-score.

Table 3 presents the within-person change for instant skin carotenoid scores and BMI outcomes for children at maintenance. Overall, the average instant skin carotenoid change was −2.0 ± 7.3 (total—groups 1, 2, and 3) for all groups, which meant that the instant skin carotenoid score decreased between post-test and maintenance. There was no maintenance data for groups 4 and 5 because of the timing of the COVID-19 pandemic. Group 1 reported an average score change of −3.4 ± 12.7 at maintenance. Group 2 had an average score change of −18.9 ± 13.6. Group 3 had an average score change of 12.2 ± 12.0 and was the only group with an increase in instant skin carotenoid score between post-test and maintenance.

**Table 3:** Descriptive statistics for within-person change in child nutrition outcomes at maintenance for Mexican-heritage children.

Outcomes	Total	Group 1	Group 2	Group 3	Group 4	Group 5	*p*-Value
Number of children, *n*	24	8	7	9	0	0	
Program dose, mean number of sessions (SD)	4.72 (2.15)	5.59 (0.94)	4.69 (2.19)	4.94 (2.12)	-	-	<0.001
Within-person change in instant skin carotenoid score	−2.00 (7.3)	−3.38 (12.71)	−18.86 (13.59)	12.22 (11.98)	-	-	0.233
Within-person change in BMI percentile	−1.6 (0.37)	−0.48 (0.64)	−2.45 (0.68)	−1.93 (0.6)	-	-	0.0925
Within-person change in BMI z-score	−0.05 (0.01)	−0.01 (0.03)	−0.07 (0.03)	−0.06 (0.02)			0.2133

BMI: Body Mass Index; SD Standard Deviation. Data for 24 children with fruit and vegetable intake and BMI outcome data at maintenance. This table presents changes in outcomes between the post-test and maintenance measures. *Promotoras* completed maintenance measures three to four months after the post-test. *p*-values are for uncorrected overall *p*-value for analysis of variance (ANOVA) for differences in mean changes between intervention and control groups. Differences were considered statistically significant at *p* < 0.05.

Regarding BMI outcomes at maintenance, the average BMI percentile change was −1.6 ± 0.37 (total for all groups), which meant that BMI percentile decreased between post-test and maintenance. Group 2 had the most noticeable decrease in BMI percentile (−2.5 ± 0.68) at maintenance. Groups 1–3 reported reductions in BMI at maintenance (three to four months later). A similar pattern was seen for BMI z-score.

### 3.3. Program effects on Instant Skin Carotenoid Scores and BMI for Children at Post-Test

Table 4 presents the overall change for instant skin carotenoid score and BMI outcomes for children at post-test. In the unadjusted models, the program decreased the instant skin carotenoid score (−14.7l, 95% CI: −30.9, 1.6) and decreased BMI percentile (−0.2, 95% CI: −2.2, 1.8) for children at post-test. A similar pattern was seen for BMI z-score. In adjusted models, the change in skin carotenoid score was strengthened and significant (−15.14; 95% CI: −24.95, −5.33). Unadjusted and adjusted estimates were similar for BMI outcomes.

## 4. Discussion

This study examined the effects of the HEPP program on instant skin carotenoid score, as a biomarker of fruit and vegetable intake, and age- and sex-adjusted BMI at post-test and maintenance. Overall, the results showed that the HEPP program had no statistically significant effects on instant skin carotenoid score or BMI for Mexican-heritage children at post-test, based on unadjusted and adjusted models. In addition, there were no statistically significant within-person changes by groups aside from dose for group 5. However, for some groups, associations were in the hypothesized direction, and there were greater effects for the program (intervention) group compared to the control group for BMI outcomes, which provides some evidence for program effectiveness.

Preliminary evidence from this program is promising. In the U.S., systemic, structural, and social factors, like poverty and food insecurity, disproportionately affect Latino communities, which means that Latino households have not had resources or opportunities to support adequate nutrition. For example, based on the U.S. Department of Agriculture’s (USDA) most recent report, food insecurity prevalence was 10.8% in Hispanic households with children compared to 3.6% in non-Hispanic white households with children in 2021 [28]. Although the sample was not nationally representative, our data showed that 68.4% of participants lived in households considered food insecure. Previous research documents how food insecurity limits access to health-promoting foods [42]. Studies with national samples of Latino/a children have reported racial/ethnic disparities in fruit and vegetable (FV) intake and BMI [28]. Overall, this program showed a null effect on a biomarker of FV intake (instant skin carotenoid status), and a reduction in BMI for children. However, given the need to support nutrition among Latino/a children, this manuscript makes an important contribution.

Findings highlighted interesting patterns between groups, likely related to group variations in program retention and timing of delivery. For example, group 3 consistently reported greater changes in outcomes compared to the other groups at post-test and maintenance. One reason for the larger effect sizes may have been gains in confidence or skill of the *promotora* group leaders/interventionists over time. Another reason may have been peer learning or social support that participants experienced in this group 3 [9,10].

Results also generated important insights related to school lunch programs. Group 1 was the only group that started and finished the program during the summer and the only group with an increase in Veggie Meter^®^ scores at post-test (and an increase relative to control group). When school was not in session, children may have had limited access to fruits and vegetables through the school meal programs and benefited more from the HEPP program. When school was in session, children likely had greater access to fruits and vegetables, through the school meal programs, including the Community Eligibility Provision [43], and increased dietary intake of fruits and vegetables, resulting in an increase in Veggie Meter^®^ score. School lunch programs have been shown to increase the selection and dietary intake of fruits and vegetables [44] in children participating in the program by up to 23% [45]. Furthermore, children between the ages of 9 to 12 years old have been found to consume over half of their daily fruit and vegetable intake while at school [46]. We expected a larger increase in Veggie Meter^®^ score for children in groups 2, 3, and 4, who participated in the HEPP program during the school year and may have had access to school lunch programs. Yet, our results showed reductions in Veggie Meter^®^ scores for some groups, which suggests that children’s access to fruits and vegetables through school lunch meals program may have been insufficient to affect instant skin carotenoid score.

A couple of points warrant additional discussion. First, the program evaluated its primary outcome of dietary intake of fruits and vegetables with the Veggie Meter^®^, which was a relatively new device at the time. Available evidence from Veggie Meter^®^ studies now indicates that the exposure period is about eight weeks [32]. However, this program lasted six weeks, and the relatively short duration may have made it more difficult to observe a change in instant skin carotenoid score. There was also tremendous variation in data from the Veggie Meter^®^. The *promotoras* documented numerous issues with residual food staining, which initiated a protocol deviation or changing the finger for assessment. Anecdotally, when the *promotoras* scanned a different finger, the score was quite different. The small sample size and large variation in Veggie Meter^®^ scores likely made it difficult to estimate effect size. To date, though, there has been limited published research on using Veggie Meter^®^ with Latino children or adults [47,48] and few studies have assessed skin carotenoids among Latino/a children [49,50]. However, based on the authors’ knowledge, there have been no published studies using the Veggie Meter^®^ with Latino/a children living in border communities and only one study that obtained Veggie Meter^®^ scans at more than one time point for a subsample of Latino/a children [51].

Based on the team’s experience, there are several practical benefits to using the Veggie Meter^®^ in community-based research. First, the Veggie Meter^®^ is a rapid assessment, and assessments only take a couple of minutes. There is no additional data entry and sources of error are reduced. Second, the Veggie Meter^®^ is a small and lightweight device and easy to transport. Third, the non-invasive nature of the assessment reduced the burden and increased the acceptability of the program, which was grounded in community-engaged research. This allowed for inadequate fruit and vegetable intake to be identified and to evaluate the success rates of intervention programs that aim at increasing fruit and vegetable intake in study participants [30]. Anecdotally, the *promotoras* shared that the Veggie Meter^®^ was quick and easy to use for them, and accepted by families, especially for children.

Second, this program evaluated BMI in growing children. Findings showed that the program resulted in a decrease in BMI outcomes at post-test and maintenance. Program effects may be attributed to the family functioning, nutrition, or the physical activity components of the program [12,13,14]. There may have been other factors affecting children’s BMI, which were unmeasured and not considered in analyses. In addition, while some programs have reported similar effects among Latino/a children, successful programs had longer durations or samples from varied racial and ethnic backgrounds, which could lead to different results [52,53]. For example, Gallo et al. examined the 10-week program Vidas Activas y Familas Saludables (VALE) and found statistically significant decreases in child BMI for age z-scores, waist circumference, and percent body fat [52]. Robinson et al. evaluated a three-year intervention program in Latino/a children and found children gained an average ~0.25 kg·m^2^ less than those in control group over the three-year period [53]. The effects at maintenance of a decrease of −0.05 in BMI z-score (−0.20 to −0.25 BMI z-score change) were insufficient for clinical significance for BMI outcomes in children [54,55]; however, the smaller sample size and imprecision of estimates made it somewhat difficult to determine effect size.

### 4.1. Limitations and Strengths

This study is not without limitations. First, the sample size was relatively small (59 children enrolled and 41 children with pre- and post-test data). The design, implementation, and evaluation required tremendous resources, which limited the number of families that were able to participate. Prior studies, family-based nutrition interventions, have included samples of between nine and 356 children [49,56,57,58,59]. A larger sample size would have improved precision of estimates and may have provided sufficient power to detect statistically significant effects. The program was relatively shorter than other programs (duration six weeks to 16 weeks) [3,7,59,60,61,62], but more comprehensive and intensive. For example, the program included family functioning, nutrition, and physical activity and was intense, with a greater dose (900 min or 15 h) compared to previous programs with a similar focus (range for dose: 10.5 to 20 h) over a shorter six-week duration (range: 6 to 12 weeks) [12]. Additional research will be needed to balance resource demands of the program with efficacy. Second, the COVID-19 pandemic negatively impacted this program and resulted in missing data at post-test for all of group 5 and missing data at maintenance for groups 4 and 5. Limited data may have biased the results for within-person and between group differences. Imputation, such as baseline observation carried forward, was not considered for this analysis because of the risk of bias associated with single imputation [63]. Multiple imputation was not considered because of the relatively small sample and limited data available for multiple imputation. Third, as with all intervention programs, it is not possible to account for and measure all the factors that might impact the program or outcomes. This study attempted to minimize threats to internal validity by including a comparison or control group, using more objective measures for outcomes, and defining outcome variables with a change score. For example, in analyses, the use of within-person change to define outcomes captures time-invariant inherent immeasurable factors associated with the outcomes and reduces between-person variability [64]. However, future research is needed to fully understand and minimize confounding bias. Fourth, relatively early adoption of a new device like the Veggie Meter^®^ meant that there was not a standard research protocol for how to use the Veggie Meter^®^ available at the time of the program. The research team overcame this barrier by developing a protocol (described in the methods) that outlined conducting scans on the right index finger for all children, unless there was a reason to use the left hand due to residual staining or “out of range” scores. However, recommendations published in 2022 suggested that scans use the non-dominant ring finger, which was not done in this study. Because of the timing, and recommended protocol being unavailable [32], findings for the instant skin carotenoid score must be interpreted carefully.

At the same time, use of the Veggie Meter^®^, as an objective measure of dietary intake, was a strength and sets this program apart. Validation studies have shown a high correlation between Veggie Meter^®^ scores and serum carotenoids, which is a more reliable way to measure fruit and vegetable intake compared to self-reported dietary recalls and food records, which are prone to inherent bias and inaccurate estimates [32,35,65]. Additional strengths were using anthropometry versus self-report to obtain height and weight [63]. This study included a maintenance measure, which is another strength. Originally, the program intended to collect maintenance measurements for all groups to determine short-term impacts at 3–4 months. Generally, programs have not collected data for maintenance and assessing outcomes at maintenance contributes valuable evidence. The need for additional research on long-term impacts has been documented in the literature to determine the sustainability of intervention effects and strengthen and improve intervention strategies [66,67,68,69]. Importantly, the strength of this program is attributed to the community-engaged approach with a *promotora* model, use of theory, and targeting family functioning to engage fathers, mothers, and children as a family system [12,13]. Additionally, this program uniquely investigated individual measures within the context of family and community systems, which underscores the value of this research.

### 4.2. Implications

Findings offer implications for practice, research, and policy. First, the Veggie Meter^®^ was a relatively easy assessment for *promotoras* and acceptable to the Mexican-heritage children. The device provided a rapid, non-invasive, and portable way to obtain an objective measure of dietary intake [32] and may be valuable in community-based office or clinical settings. However, the relatively high cost of a Veggie Meter^®^ may make it difficult for community-based organizations or researchers to acquire. Using the Veggie Meter^®^ for research offered several advantages, including using an objective measure to evaluate the program, portability, and minimizing sources of error in data entry. However, future research is needed to create standardized protocols or best practices for research with the Veggie Meter^®^ and create a data repository of Veggie Meter^®^ scores for different subpopulations, including children from different racial and ethnic backgrounds, ages, and genders. Protocols will support validity and reliability of findings and a data repository will help with data interpretation, especially for special populations, including children. Given that the Veggie Meter^®^ reflects a reference period of about eight weeks [32], this assessment may be better suited for programs with a longer duration (eight weeks or longer). Second, the promising results were likely related to the community-engaged approach with a *promotora* model and theory-based design [12,13]. The approach led to an effective program that may have been culturally meaningful to families and individual children. Future research is needed to understand which components of the program were efficacious, and specifically, a process evaluation is required before considering replication or adaptation. Because this family-centered program targeted family functioning, data were collected on family functioning with a validated scale [12]. Additional analysis may provide important insights into effects on family functioning or how family functioning related to outcomes, if at all. Future research is needed to test this program with a larger sample. Lastly, findings have policy relevance and novel approaches that may support nutrition security. The USDA has defined nutrition security as having consistent and equitable access to healthy, safe, affordable foods essential to optimal health and well-being [70]. There may be a need to strengthen school meal programs, and emphasize carotenoid-rich fruit and vegetable intake, especially for communities with limited access.

## 5. Conclusions

This study applied an inclusive approach to behavioral nutrition and targeted family functioning for Mexican-heritage families living in the Lower Rio Grande Valley of Texas. While the program was not a weight-centered program, there were promising results for BMI at post-test for Mexican-heritage children (six weeks after baseline or program start) and somewhat sustained effects on BMI at maintenance (three to four months after program completion). However, the *promotoras* documented numerous issues with residual food staining that affected the consistency of the Veggie Meter^®^ scans, and results showed that the program did not have a positive effect on instant skin carotenoid score at post-test or maintenance for children across groups. Additional research can help establish protocols for collecting and interpreting Veggie Meter^®^ data with Latino/a children [48]. Our findings provide evidence for strengths-based approaches in behavioral nutrition and strengthening federal nutrition and food assistance programs, like the National School Lunch Program, which have been shown to increase fruit and vegetable intake among children and support food security for their families [68,69].

## Figures and Tables

**Table 1:** Baseline characteristics of full sample of Mexican-heritage children in the HEPP program.

Characteristics	Total	Group 1	Group 2	Group 3	Group 4	Group 5	*p*-Value
Number of children, *n*	57	12	10	12	12	11	
Child							
Mexican-heritage ethnicity	57 (100%)	9 (100%)	10 (100%)	12 (100%)	12 (100%)	11 (100%)	
Age	10.21 ± 0.94	10.26 ± 0.27	10.36 ± 0.3	10.1 ± 0.27	10.52 ± 0.27	9.8 ± 0.28	
**Sex**							0.203
Male	27 (47.37%)	5 (41.67%)	3 (30%)	9 (75%)	4 (33.33%)	6 (54.55%)	
Female	30 (52.63%)	7 (58.33%)	7 (70%)	3 (25%)	8 (66.67%)	5 (45.45%)	
Instant skin carotenoid score (biomarker for dietary intake of fruits and vegetables)	213.54 ± 61.57	242.63 ± 17.77	208.7 ± 19.46	213.79 ± 17.77	194.46 ± 17.77	206.77 ± 18.56	
BMI z-score	1.14 ± 1.15	0.9 ± 0.34	1.05 ± 0.37	1.2 ± 0.34	1.35 ± 0.34	1.21 ± 0.36	
BMI percentile	77.57 ± 27.89	69.04 ± 8.21	74.26 ± 8.99	81.24 ± 8.21	82.84 ± 8.21	80.12 ± 8.57	
**BMI categories**							0.997
<85th percentile	22 (38.6%)	6 (50%)	4 (40%)	4 (33.33%)	4 (33.33%)	4 (36.36%)	
85th to <95th percentile	11 (19.3%)	2 (16.67%)	2 (20%)	2 (16.67%)	3 (25%)	2 (18.18%)	
≥95th percentile	24 (42.11%)	4 (33.33%)	4 (40%)	6 (50%)	5 (45.45%)	5 (45.45%)	
**Household food insecurity**							0.154
Food Secure	18 (31.58%)	3 (25%)	5 (50%)	2 (16.67%)	2 (16.67%)	6 (54.55%)	
Food Insecure	39 (68.42%)	9 (75%)	5 (50%)	10 (83.33%)	10 (83.33%)	5 (45.45%)	

BMI: Body Mass Index; Data for 57 children excluding two children with missing pre-test measures. *Promotoras* collected baseline data at the pre-test measure. *p*-values are for chi^2^ tests of the unadjusted percentage distributions of categorical covariates and uncorrected overall *p*-value for analysis of variance (ANOVA) for means of continuous covariates. Differences were considered statistically significant at *p* < 0.05.

**Table 4:** Program effects on instant skin carotenoid score and BMI for Mexican-heritage children.

	Unadjusted Models		Adjusted Models	
Outcome	Estimate	(95% CI)	Estimate	(95% CI)
Change in instant skin carotenoid score	−14.67	(−30.9, 1.56)	−15.14	(−24.95, −5.33)
Change in BMI percentile	−0.18	(−2.16, 1.79)	−0.20	(−0.89, 0.49)
Change in BMI z-score	−0.02	(−0.1, 0.06)	−0.02	(−0.05, 0.00)

BMI: Body Mass Index; CI: Confidence Interval. This table presents model estimates and mean predicted values and 95% confidence intervals (95% CI) for fruit and vegetable intake or BMI outcomes, based on change scores between pre- and post-test. Data for 41 children excluding 18 children missing pre- or post-test data. Estimates are mean within-person changes in outcomes obtained using Stata’s margins command. The adjusted models controlled for age, sex, month of baseline data collection, intervention/program dose (number of sessions attended), and baseline value of outcome.

## Data Availability

The data presented in this study are not available to share. Analyses are ongoing and findings will be presented in forthcoming manuscripts.

## Supplementary Materials

The following supporting information can be downloaded at: https://www.mdpi.com/article/10.3390/nu15071600/s1. Table S1. Program Completion by Group; Table S2. Protocol Deviations, Out of Range Scores, and Range of Veggie Meter^®^ Scores by Measurement Visit.
