# Supplementary material for: Nutrition Effects of a Family-Centered Health Promotion Program for Mexican-Heritage Children in the Lower Rio Grande Valley of Texas
Source: Nutrients. 2023 Mar 25;15(7):1600. doi: 10.3390/nu15071600 (PMC10097021; doi:10.3390/nu15071600)
Supplement: Supplementary file 1 [file nutrients-15-01600-s001.zip › nutrients-2291852-supplementary.pdf]

Supplementary Table S1. Program completion (number of sessions) by group.

**Completion**	**Total (n = 59)**	**Group 1 (n = 12)**	**Group 2 (n = 10)**	**Group 3 (n = 13)**	**Group 4 (n = 12)**	**Group 5 (n = 12)**
All, 6 sessions, n (%)	31 (52.5%)	7 (58.3%)	5 (50%)	9 (69.2%)	10 (83.3%)	0
Most, 4 or 5 sessions, n (%)	4 (6.8%)	1 (8.3%)	2 (20%)	0	1 (8.3%)	0
Half, 3 sessions, n (%)	2 (3.4%)	1 (8.3%)	0	1 (7.7%)	0	0
Some, 1 or 2 sessions, n (%)	13 (22.0%)	2 (16.7%)	1 (10%)	1 (7.7%)	0	9 (75%)
None, no sessions, n (%)	9 (15.3%)	1 (8.3%)	2 (20%)	2 (15.4%)	1 (8.3%)	3 (25%)

This table presents counts and proportions for program completion. The program had six weekly sessions. Children in groups 1 through 4 completed a maximum of six sessions. Due to the timing of the COVID-19 pandemic in winter of 2020, group 5 stopped at session 2. Children in group 5 completed a maximum of two sessions.

Supplementary Table S2. Protocol deviations, out of range Veggie Meter® scores, and range of scores by measurement visit.

	**Transition**	**Pre-test**	**Post-test**	**Maintenance**
Number of protocol deviations	6	7	14	0
Number of out-of-range Veggie Meter® scores	5	4	8	1
Number of missing scores (out-of-range error with Veggie Meter®)	1	2	3	0
Minimum–maximum score	52– 341	17–409	24–498	34– 428
Number of scores below 100	4	7	10	1
Example comments from promotora	“Used left index finger because the right index finger had hot Cheetos residue and reading was showing out of range.”	“Child had been eating Cheetos and had to use other hand.”	“When measurement for right index finger was conducted reading was out of range. Second try on left index finger reading was also out of range.”	“Out of range twice, switched to the left index finger.”

The HEPP program included assessments at transition, pre-test, post-test, and maintenance. Protocol deviations were documented for the following reasons: error that score was “out of range” or observations that child had residual staining from

foods or cuts on finger. An out of range error meant that there was no instant skin carotenoid score from the Veggie Meter® scan, and score was missing. In some cases, a promotora was not able to obtain a reading on the first attempt or second attempt (even after recalibrating), and after a failed second attempt, they obtained a score on the other finger and documented that score. Promotoras noted when which finger was used for the scan, they received errors of out of range, and they made multiple attempts to obtain a Veggie Meter® scan (to obtain instant skin carotenoid score).
